# Patient-specific simulation of stent-graft deployment in type B aortic dissection: model development and validation

**DOI:** 10.1007/s10237-021-01504-x

**Published:** 2021-08-24

**Authors:** Xiaoxin Kan, Tao Ma, Jing Lin, Lu Wang, Zhihui Dong, Xiao Yun Xu

**Affiliations:** 1 Department of Chemical Engineering, Imperial College London, London, SW7 2AZ UK; 2 Department of Vascular Surgery, Zhongshan Hospital, Fudan University, Shanghai, China; 3 Key Laboratory of Textile Science and Technology of Ministry of Education, College of Textiles, Donghua University, Shanghai, China

**Keywords:** Finite element analysis, TEVAR, Type B aortic dissection, Virtual stent-graft deployment

## Abstract

Thoracic endovascular aortic repair (TEVAR) has been accepted as the mainstream treatment for type B aortic dissection, but post-TEVAR biomechanical-related complications are still a major drawback. Unfortunately, the stent-graft (SG) configuration after implantation and biomechanical interactions between the SG and local aorta are usually unknown prior to a TEVAR procedure. The ability to obtain such information via personalised computational simulation would greatly assist clinicians in pre-surgical planning. In this study, a virtual SG deployment simulation framework was developed for the treatment for a complicated aortic dissection case. It incorporates patient-specific anatomical information based on pre-TEVAR CT angiographic images, details of the SG design and the mechanical properties of the stent wire, graft and dissected aorta. Hyperelastic material parameters for the aortic wall were determined based on uniaxial tensile testing performed on aortic tissue samples taken from type B aortic dissection patients. Pre-stress conditions of the aortic wall and the action of blood pressure were also accounted for. The simulated post-TEVAR configuration was compared with follow-up CT scans, demonstrating good agreement with mean deviations of 5.8% in local open area and 4.6 mm in stent strut position. Deployment of the SG increased the maximum principal stress by 24.30 kPa in the narrowed true lumen but reduced the stress by 31.38 kPa in the entry tear region where there was an aneurysmal expansion. Comparisons of simulation results with different levels of model complexity suggested that pre-stress of the aortic wall and blood pressure inside the SG should be included in order to accurately predict the deformation of the deployed SG.

## Introduction

Aortic dissection is a catastrophic aortic disease which initiates with a tear in the intimal layer of the aortic wall, through which blood flows into the medial layer, separating the intima and adventitia and forming a new flow channel known as false lumen (FL). Aortic dissection with a primary tear in the descending aorta is classified as Stanford Type B aortic dissection (Nienaber and Clough 2015). Thoracic endovascular aortic repair (TEVAR) has been recognised as a standard treatment to type B aortic dissection. Although great success has been achieved by TEVAR procedure with its advantage being less invasive, there are still unpredictable procedure-related complications, such as stent-graft-induced new entry (SINE) and retrograded type A aortic dissection (RTAD) (Dong et al. 2010; Dong et al. 2009).

In the past decade, clinical studies have investigated the risk factors responsible for these TEVAR complications (Dong et al. 2010; Dong et al. 2009). Earlier generations of stent-graft (SG) products were shorter in length. They consist of multiple metal struts and a vertical connecting bar to prevent the SG from excessive twisting. This design was believed to generate extra spring-back force as the SG has an inherent tendency of recovering to its originally straight status after been deployed into a curved aorta, which could lead to stress concentration in the aortic wall and injury to the intima.(Dong et al. 2010). The SG devices shorter than 165 mm were found to have a high SINE incidence at the distal end due to excessive spring-back force after the implantation in curved descending aorta (Ma et al. 2018a). The SG diameter relative to the local aorta diameter, also known as the oversizing ratio, is regarded as another key risk factor for SINE and RTAD (Canaud et al. 2019; Ma et al. 2018a; Pantaleo et al. 2016). These SG configuration and SG/aorta mechanical interaction are crucial to the post-TEVAR biomechanical behaviour and consequential clinical results. However, current pre-surgical planning and SG selection are based more on anatomical geometries and clinical guidelines, but less on quantitative biomechanical analysis and risk assessment (Pape et al. 2015). Neither biomechanical forces, such as SG-induced spring-back force and radial force, nor the SG final configuration are available to clinicians when making pre-surgical decisions.

Several finite element method (FEM)-based numerical simulation studies have been published focusing on SG deployment in the aorta. In 2012, De Bock et al. reported their SG deployment simulation in an idealised abdominal aortic aneurysm model, demonstrating a good agreement with the corresponding in *vitro* stenting experiment (De Bock et al. 2012). Auricchio et al. were the first to report simulations of SG deployment in a patient-specific ascending aorta, although the aorta was assumed to be rigid (Auricchio et al. 2013). This was extended to compliant abdominal aorta models by Perrin et al. who demonstrated a robust morphing methodology to deploy a tubular SG into complex aortic geometry (Perrin et al. 2016, 2015a, 2015b). Other relevant studies examined different aspects of SG deployment simulation, such as the deployment process, more complex SG morphology and the consideration of blood flow (Derycke et al. 2019; Romarowski et al. 2018, 2019). Simulations of SG deployment in abdominal aortic aneurysms using an in-house code has also been reported, demonstrating high robustness and efficiency (Hemmler et al. 2019a, 2019b, 2018).

However, patient-specific simulation of SG deployment in type B aortic dissection poses additional challenges, due to the complex aortic anatomical geometry, such as the presence of multiple tears, narrowed true lumen and highly tortuous false lumen. Therefore, FEM-based simulation of SG deployment in patient-specific aortic dissection models requires accurate control of the SG movement. To our best knowledge, only two FEM-based simulations of SG deployment in type B aortic dissection have been reported so far (Ma et al. 2018b; Meng et al. 2020), but these studies did not consider the effect of pre-stress in the aortic wall nor blood pressure. On the other hand, using FEM to calculate the pre-stress field corresponding to diastolic blood pressure has been applied to the simulation of aortic root and stent deployment in aortic coarctation (Caimi et al. 2020; Votta et al. 2017).

In this study, we present a FEM-based simulation method for SG deployment in a patient-specific type B aortic dissection model. Pre-stress of the aortic wall and intraluminal blood pressure were incorporated and their influences on the simulation results were examined. The material parameters for the aortic wall were fitted to the tensile testing data of descending aortic wall samples taken from type B aortic dissection patients. In what follows, we will describe the geometric reconstruction approach and simulation framework for SG deployment in type B aortic dissection. Simulation results obtained with different levels of model complexity will then be compared with post-TEVAR imaging data to assess the model accuracy.

## Methodology

### Patient-specific type B aortic dissection geometry

**Fig. 1:**
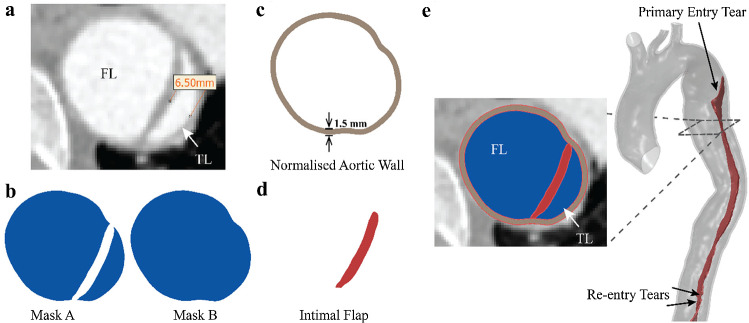
Illustration of the segmentation and reconstruction of aortic dissection geometry from CTA scan. **a** The transverse view of the descending aorta before TEVAR showed true lumen (TL), false lumen (FL) and the measurement of narrowed true lumen. **b** The segmentation mask A represents the blood flow domain and mask B encloses the flow domain and the intimal falp. **c** The aortic wall was created by extruding mask B outwardly by 1.5 mm. **d** The segmentation of intimal flap was created by performing Boolean subtraction of mask A from mask B. **e** The aortic wall and intimal flap were combined and trimmed to form the pre-TEVAR aortic dissection geometry

A 44-year-old female complicated type B aortic dissection patient with a narrowed true lumen, who underwent TEVAR procedure at sub-acute phase, was included in this study. The study complied with the Declaration of Helsinki and was approved by the ethics committee of Zhongshan Hospital, Fudan University. All patients provided written informed consent for participation. Computed tomography angiography (CTA) scans of the patient were acquired before TEVAR (Fig. 1.a) and 3 months after TEVAR. CTA scans were performed with Aquilion ONE (Toshiba Medical Systems, Otawara, Japan) (1.0 mm slice thickness and 0.68 mm × 0.68 mm pixel size). The compressed true lumen had a local diameter of 6.5 mm at the narrowest section.

The CTA images were processed in Mimics 22.0 (Materialise, Leuven) software. Two masks were first segmented from the pre-TEVAR CTA: the segmentation mask A included the blood flow domain of the true and false lumen, while the segmentation mask B enclosed the true and false lumen and the intimal flap (Fig. 1b). The aortic wall was created from mask B which was extruded outwardly by a uniform thickness of 1.5 mm (Fig. 1c). Boolean calculation was then performed by subtracting mask A from mask B to obtain the segmentation of intimal flap (Fig. 1d). Therefore, the distance between the true and false lumen in mask A represented the local thickness of the intimal flap which was in the range of 0.6–1.6 mm in this patient. The geometry of the aortic wall and intimal flap were smoothed and combined using Meshmixer (Autodesk, Inc.). The proximal and distal ends of the dissection model were trimmed by placing cutting planes perpendicular to the local centreline in the proximal ascending aorta, distal segments of the supra-aortic branches and in the abdominal aorta above the aortic bifurcation (Fig. 1e). The intimal flap and the aortic wall were meshed with tetrahedral elements (C3D4 in Abaqus® (Dassault Systèmes, France)).

### Mechanical properties of dissected aorta and intimal flap

The histopathology of intimal flap changes as aortic dissection progresses from acute phase to chronic phase, resulting in significantly different mechanical behaviour (Peterss et al. 2016). However, very little information is available on the mechanical behaviour of intimal flap in different phases of the dissection. A recent study has shown that the intimal flap exhibits linear elastic response rather than nonlinear behaviour seen in healthy aorta (Deplano et al. 2019). Therefore, in this study the intimal flap was modelled as a linear elastic material with a Young’s modulus of 277 kPa and Poisson’s ratio of 0.49 (Deplano et al. 2019).

In order to obtain material properties representative of aortic dissection, twelve tissue samples were obtained from 5 type B aortic dissection patients who underwent open surgery for descending aorta replacement in Zhongshan Hospital, Shanghai, China. The tissue samples were cut into strips (40 × 10 mm) along the circumferential direction, refrigerated at 4℃ in 0.9% NaCl solution and tested within 72 h after the surgery. Uniaxial tensile tests were performed by using the YG(B)026G-500 Electronic Tensile Testing System (Darong Textile Instrument Co., Ltd, Wenzhou, China) (load cell accuracy 0.01 N; step motor resolution 1 µm). The initial distance between the two grips was set as 25 mm and a preconditioning load of 0.05 N was applied to the samples. The tensile tests were performed with a displacement rate of 1.6 mm/s until the tissue samples failed. The stretch ratio λ was calculated from the distance between the two grips. With the incompressible assumption for aortic tissue (Holzapfel 2000), the Cauchy stress can be calculated from the measured force, the strip thickness and the stretch ratio.

The Yeoh strain energy function was employed to characterise the mechanical behaviour of the type B aortic dissection tissue samples (Yeoh 1993).

$$W={c}_{10}\left({\overline{I} }_{1}-3\right)+{c}_{20}{\left({\overline{I} }_{1}-3\right)}^{2}+{c}_{30}{\left({\overline{I} }_{1}-3\right)}^{3}$$

where ${c}_{10}$, ${c}_{20}$, ${c}_{30}$ are material parameters, ${\overline{I} }_{1}$ is the first deviatoric invariant defined as

$${\overline{I} }_{1}= {\overline{\lambda }}_{1}^{2}+{\overline{\lambda }}_{2}^{2}{+\overline{\lambda }}_{3}^{2}$$

where the deviatoric stretch ratios ${\overline{\lambda }}_{i}={J}^{-1/3}{\lambda }_{i}$, $i=\mathrm{1,2},3$, being $J$ the total volume ratio and ${\lambda }_{i}$ the principal stretches. Each stress–stretch curve of the test was fitted from zero stretch to the yield point by using an in-house MATLAB code (MathWorks, Inc., Natick, USA) to determine a set of material parameters. Values for material parameters were obtained for each tissue sample, which were then averaged and the mean values were employed to describe the incompressible hyperelastic mechanical behaviour of the aortic wall in the simulation model.

### Stent-graft modelling

**Fig. 2:**
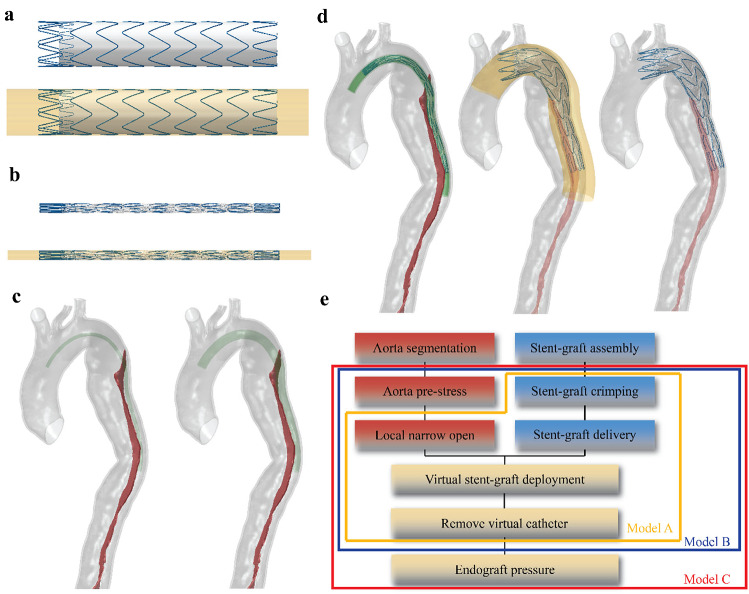
Summary of the steps in the simulation of stent-graft (SG) deployment and model variations. **a** The 28–28-150 mm Medtronic Valiant SG was used in TEVAR procedure and was covered by the virtual sheath. **b** The SG was compressed by the virtual sheath to its crimped state. **c** A curved tube opened up the local narrowing in the compressed true lumen. **d** The SG was delivered and deployed at the targeted position. **e** overall workflow and model variations

During TEVAR, a 28–28-150 mm Medtronic Valiant SG (Medtronic Vascular, Santa Rosa, California) with proximal bare metal stent was implanted. The SG geometry was created in Solidworks (Dassault Systèmes, France) following the dimension and specification of the Valliant product which consists of a Nitinol stent scaffold and polyethylene terephthalate (PET) fabric graft (Fig. 2a). The Nitinol stent was meshed into linear hexahedral elements with reduced integration (C3D8R) in Abaqus®. A superelastic material property was used, to reproduce the mechanical behaviour of Nitinol with parameters shown in Table 1 (Kleinstreuer et al. 2008). PET fabric graft was modelled as a tube with 0.1 mm thickness and meshed into membrane elements with reduced integration (M3D4R). The material property of PET fabric was simplified by assuming it as an isotropic elastic material with parameters taken from the same study (Kleinstreuer et al. 2008).

**Table 1:** Superelastic material parameters for Nitinol (Kleinstreuer et al. 2008)

Austenite elastic modulus ${E}_{\mathrm{A}}$, MPa	51,700
Austenite Poisson’s ratio ${\nu }_{\mathrm{A}}$	0.3
Martensite elastic modulus ${E}_{\mathrm{M}}$, MPa	47,800
Martensite Poisson’s ratio ${\nu }_{\mathrm{M}}$	0.3
Transformation strain	0.063
Start of transformation (loading) ${\sigma }_{\mathrm{L}}^{\mathrm{s}}$, MPa	600
End of transformation (loading) ${\sigma }_{\mathrm{L}}^{E}$, MPa	670
Start of transformation (unloading) ${\sigma }_{\mathrm{U}}^{\mathrm{s}}$, MPa	288
End of transformation (unloading) ${\sigma }_{\mathrm{U}}^{\mathrm{E}}$, MPa	254
Start of transformation stress in compress ${\sigma }_{\mathrm{CL}}^{\mathrm{S}}$, MPa	900
Reference temperature $T$, ℃	37
Density $\rho$, g/cm^3^	6.5

The Nitinol stent and PET fabric graft were then assembled together by using the tie constraint which prevents sliding or separation of the two components. A tubular surface with a diameter of 29 mm was created outside of the SG and meshed into surface elements (SMF3D4R); this represented the virtual delivery sheath and was employed to crimp and deliver the SG into the aortic dissection.

### Numerical simulation

After aortic segmentation and SG assembly, the virtual SG deployment simulation was carried out by using the commercial finite element solver Abaqus® Explicit 2019 (Dassault Systèmes, France). The workflow is summarised in Fig. 2e.

#### Pre-stress of the aortic dissection model

Several assumptions were made when calculating pre-stress conditions in the aortic dissection model. The intraluminal blood pressure was assumed to be constant at 80 mmHg (typical diastolic pressure) and uniform in both the true lumen and false lumen. The movement of the aortic root was neglected, and all the proximal and distal ends of the model were clamped with zero displacement in all directions. To account for tethering of the aorta to its surrounding biological tissues and organs, Rayleigh damping was applied to prevent the aorta from rigid body movement. The attachment of the descending aorta to the spine was modelled by specifying four pairs of fixed spots along the descending aorta representing the intercostal arteries.

Pre-stress conditions in the aorta were calculated by following the method reported by Votta et al. (Votta et al. 2017). By using a pre-defined Cauchy stress tensor as the initial condition in Abaqus, this method involved the following steps:

1. The aorta geometry reconstructed from the pre-TEVAR CT scan was defined as ${\boldsymbol{\Omega }}_{CT}$. A uniform internal pressure normal to the internal surface of the aorta was applied, which was gradually increased from 0 to 80 mmHg, resulting in deformed configuration ${\boldsymbol{\Omega }}_{1}$ and stress tensor ${{\varvec{S}}}_{1}$.
2. Having obtained the first end diastolic stress field, the initial stress tensor was updated with ${{{\varvec{S}}}_{0}={\varvec{S}}}_{1}$ and n = 1. An iterative loop was then started with the same internal pressure loading to produce updated configuration and stress tensor, ${\boldsymbol{\Omega }}_{n+1}$ and ${{\varvec{S}}}_{n+1}$, respectively. The iteration continued until the difference between the deformed configuration under 80 mmHg pressure loading ( ${\boldsymbol{\Omega }}_{n+1}$) and the geometry reconstructed from the CT scan (${\boldsymbol{\Omega }}_{cT}$) was less than 0.5 mm.
3. Set the converged stress tensor ${{\varvec{S}}}_{n+1}$ as ${{\varvec{S}}}_{0}$ which was mapped directly onto ${\boldsymbol{\Omega }}_{CT}$.

By following this approach, the pre-stress tensor field ${{\varvec{S}}}_{0}$ corresponding to the diastolic phase was obtained. This was then applied as the initial stress in the virtual SG deployment simulation.

#### Virtual stent-graft deployment

Simulation of SG deployment including SG crimping, delivery and release, and it was performed within the reconstructed pre-TEVAR aorta geometry by applying a nodal specific displacement boundary condition on the virtual sheath. The centreline of the true lumen in the pre-TEVAR geometry was extracted and the guide wire was assumed to follow the centreline, allowing determination of the proximal landing and distal positions during SG implantation. Contacts between objects were modelled using the general contact formulation with penalty method in Abaqus Explicit.

The first step involved assembly of the SG at stress-free state, the pre-TEVAR aorta model and the virtual delivery sheath while neglecting any contact relations between the SG and aorta. This was followed by shrinking a tubular virtual sheath with an initial diameter of 29 to 7 mm to simulate the SG delivery system. The SG within this virtual sheath was compressed from its stress-free state to crimped state (Fig. 2b). Secondly, a curved tube following the centre line of the pre-TEVAR aorta was placed in the true lumen and expanded from 6 to 8 mm to open up the local narrowing in the compressed true lumen (Fig. 2c). It was assumed that there was no friction between the curved tube and aorta internal surface to avoid creating additional tangential force. The targeted proximal landing position was selected on the centre line by referring to the post-TEVAR CT scan. The target shape of the virtual sheath was then created through another virtual tube with a diameter of 7 mm along the centre line. The SG was delivered into the true lumen by deforming the virtual sheath to the target shape with the use of nodal specific displacement in three sub-steps (Fig. 2d). No sliding between the SG and virtual sheath was permitted during this process. Finally, with the crimped SG in the true lumen, the sliding restriction between the SG and virtual sheath was lifted, while the contact between the SG and inner surface of the aorta was activated with a friction coefficient of 0.1 (Vad et al. 2010). The SG was deployed by expanding the virtual sheath radially to a diameter larger than the local diameter of the aorta (Fig. 2d). The entire SG deployment simulation was completed when the SG was fully in contact with the aorta and reached mechanical equilibrium. The kinetic and internal strain energy ratio (ALLKE/ALLIE) was monitored and kept within 10% to ensure the simulation as a quasi-static analysis.

#### Change in loading condition after stent-graft deployment

After the SG was deployed into the true lumen, the primary entry tear was sealed, and blood flow was restored through the conduit formed by the SG. In the stented segment, the graft fabric and metallic stent wires shielded the aortic wall and initial flap from the pulsating blood pressure. This required a change in loading condition so that the blood pressure acting on the aortic internal surface was reduced from 80 mmHg to 0, while the graft internal pressure was increased from 0 to 80 mmHg. If blood pressure inside the graft was neglected, the graft fabric would tend to fold inwards leaving small gaps between the graft fabric and local aortic wall after SG deployment (Hemmler et al. 2019b).

### Model assessment and validation

To investigate the effect of model complexity on the simulation result, three models with and without accounting for pre-stress and the action of blood pressure were tested and compared. In model A, the effect of pre-stress in the aortic wall was neglected, and the SG was not loaded with internal blood pressure. In model B, the pre-stress tensor ${{\varvec{S}}}_{0}$ was calculated and applied as the initial stress field in the aorta, but the action of blood pressure on SG was not included. In model C, both the effect of pre-stress and the action of internal pressure on SG were considered (Fig. 2e).

**Fig. 3:**
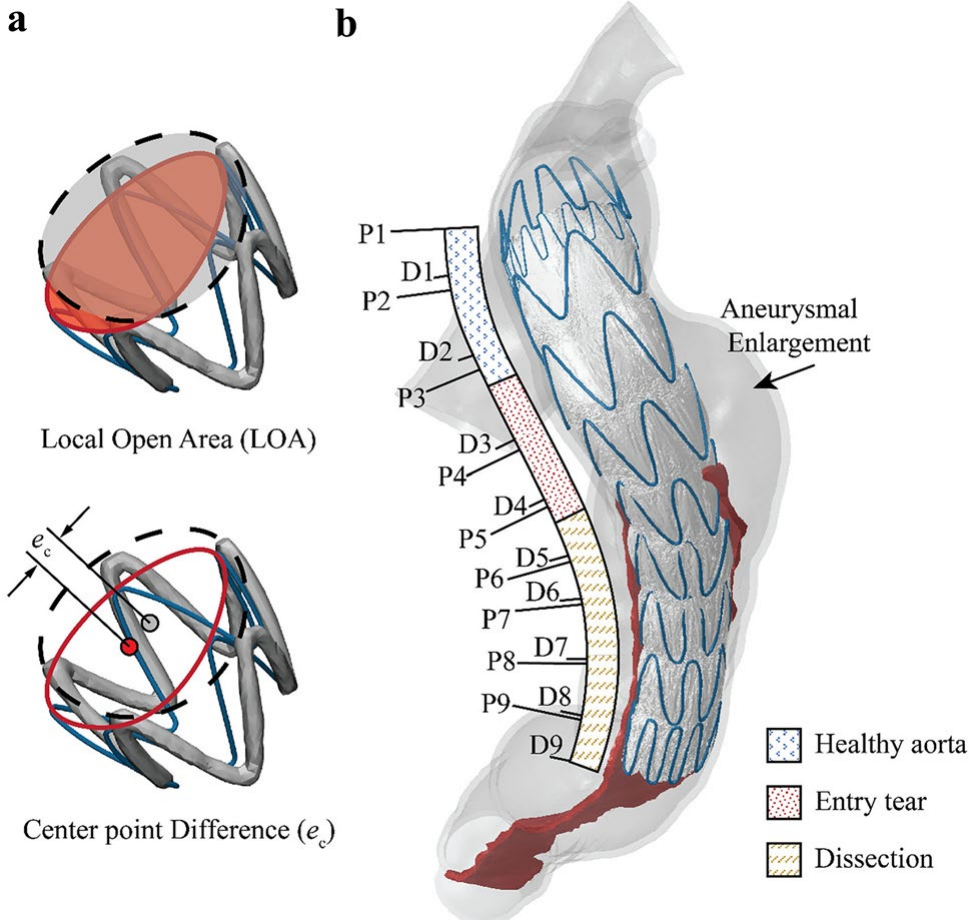
**a** Definitions of parameters for quantitative assessment of stent-graft configuration. **b** Numbering of stent strut ends and the definition of landing sections

The SG configuration reconstructed from the follow-up CT scan was used to validate the simulation results by following a similar approach adopted by others (Derycke et al. 2019; Perrin et al. 2016, 2015a). The segmented geometries from the pre- and post-TEVAR scans were registered onto the same coordinate system by using the reference points picked manually on the T4 and T11 thoracic vertebrae in Mimics 22.0. Due to the narrowed true lumen in our simulation case, the SG was compressed to a non-cylindrical configuration which cannot be assessed using the cylinder fitting approach employed in previous studies (Derycke et al. 2019). Instead, a spline was created through the apexes at each stent strut end by using SpaceClaim (ANSYS Inc., Pittsburgh, PA). The spline enclosed area was used to evaluate the local opening area (LOA) at the stent strut end. The distance between the centre points of each strut end and follow-up stent end was used to assess the strut spatial position difference (${e}_{c}$) (Fig. 3).

## Results

The material parameters for the hyperelastic behaviour of the aortic wall were obtained by fitting to the uniaxial tensile testing data on aortic tissues from type B aortic dissection patients. The SG was successfully deployed into the narrowed true lumen in all simulation models. Following the completion of each simulation, the predicted SG configuration and aortic wall biomechanics were analysed and compared.

### Material parameters for descending aortic wall in type B aortic dissection patients

**Fig. 4:**
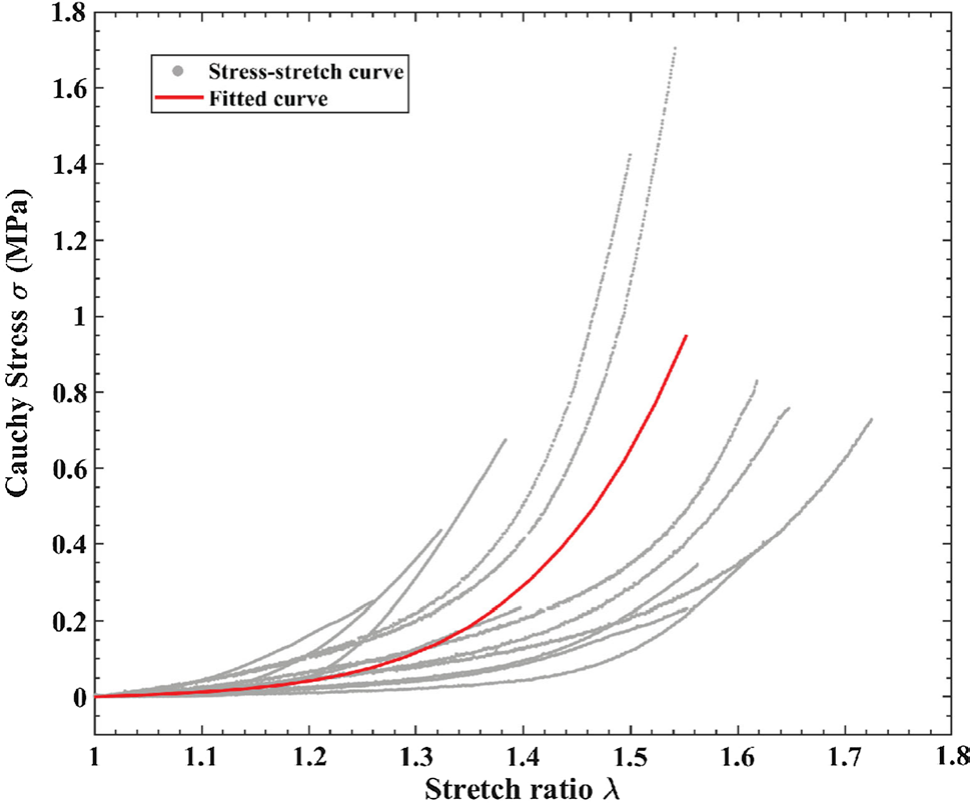
Stress–stretch relationships for aortic tissue samples taken from type B aortic dissection patients

Figure 4 shows the stress–stretch relationship for the aortic tissues from type B aortic dissection patients (*n* = 12), along with the averaged fitting curve. The coefficients of determination ${R}^{2}$, assessing the quality of fitting for the samples, had a mean value of 0.996. The averaged parameters for Yeoh model were calculated as ${c}_{10}$ = 17.5 kPa, ${c}_{20}$ = 58.9 kPa, ${c}_{30}$ = 116.1 kPa which were used in all simulations.

### SG configuration

**Fig. 5:**
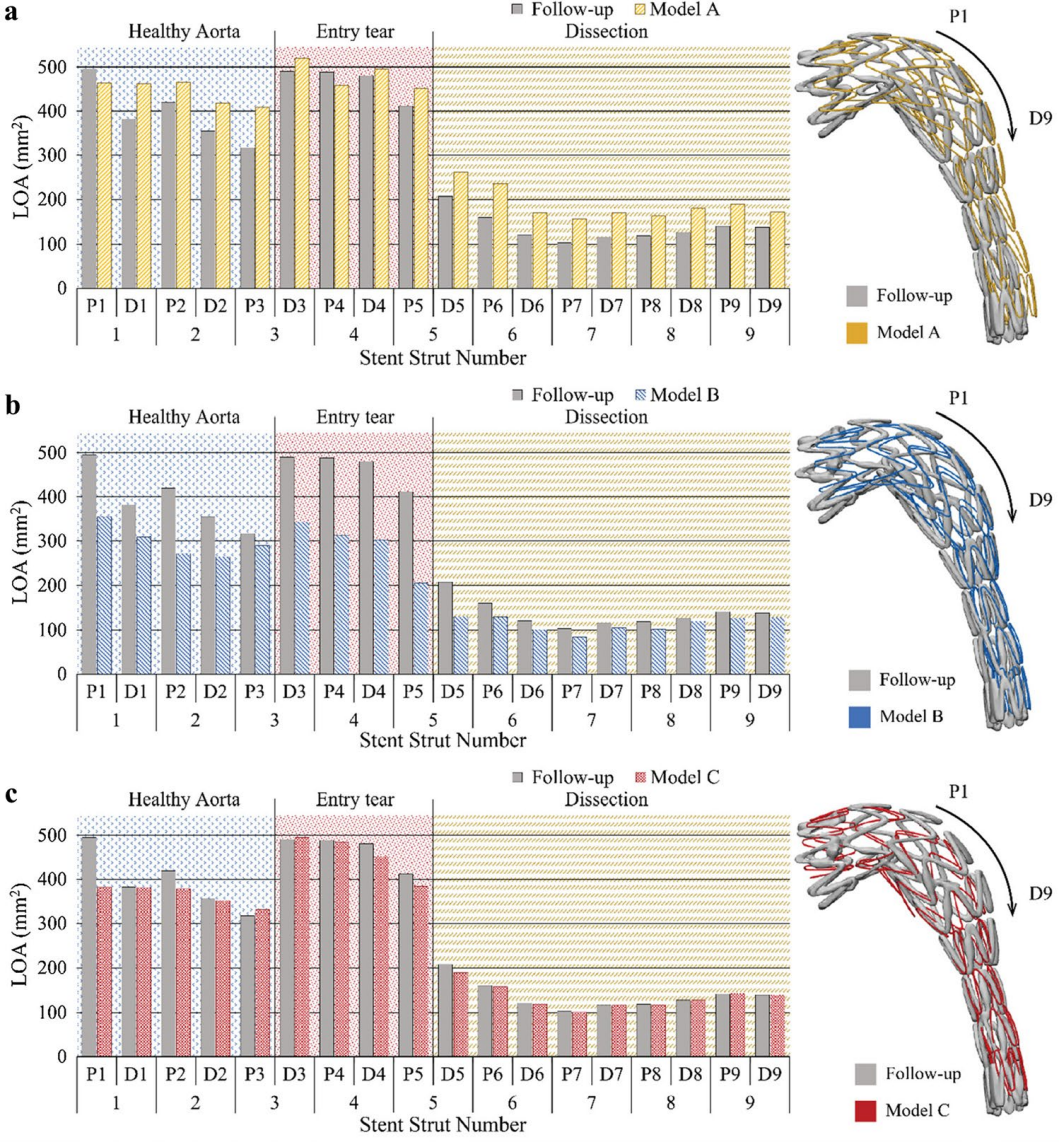
Comparisons of local open area (LOA) at the strut ends measured from model A (**a**), model B (**b**) and model C (**c**) against the corresponding values measured from the post-TEVAR follow-up CT scan

The LOA at the ends of each strut was evaluated and compared with the corresponding value obtained from the SG configuration based on the post-TEVAR CTA. As shown in Fig. 3, the stent struts are numbered from 1 to 9, with each strut having a proximal end (marked as P) and a distal end (marked as D). The nine struts landed in three landing zones according to the anatomical structure. Struts 1 and 2 and the proximal end of strut 3 (P3) landed in the healthy aorta; the distal end of strut 3 (D3), strut 4 and the proximal end of strut 5 (P5) landed in the entry tear region, while the distal end of strut 5 (D5) and the rest of the struts were in the true lumen of the dissected aorta. Based on the SG configuration reconstructed from the follow-up CT scan, it can be observed that the D3 to P5 ends had significantly larger LOA (468.0 ± 37.7 mm^2^) than the LOA of stent ends landed in other two sections, while stent struts landed in the dissection section (D5 to D9) were compressed with LOA at 137.3 ± 31.1 mm^2^. There was a dramatic change in LOA for stent struts landing on section borders, e.g. the LOA difference was 173.6 mm^2^ for P3 and D3 and 204.1 mm^2^ for P5 and D5 (Fig. 5a).

**Fig. 6:**
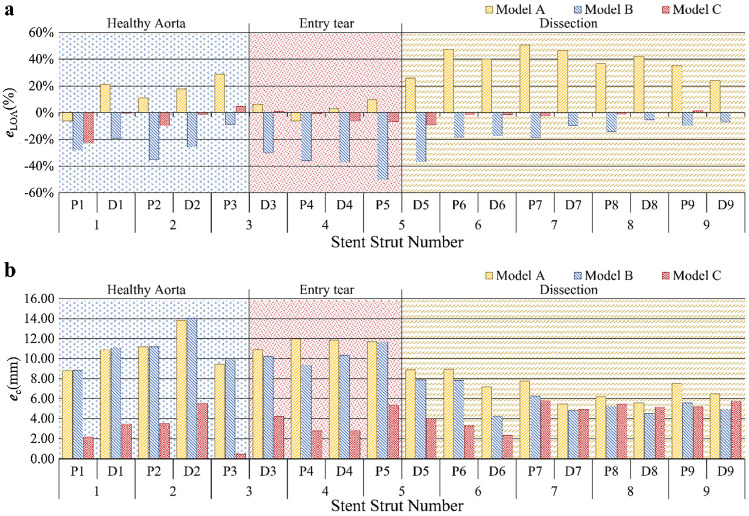
Deviations of the predicted stent-graft configuration from the three models. **a** Local open area (LOA) deviation (*e*_LOA_). **b** Stent strut centre points position deviation (*e*_c_(mm))

Results presented in Fig. 6a and Table 2 show that model A overpredicted LOA by an average of 14.5% in the healthy aorta section and 38.7% in the dissection section, with a slight overprediction of 3.2% in the entry tear region. It partially captured the significant change in LOA for stent struts landed on section borders (P3 to D3) as measured on the post-TEVAR CT scan, with LOA differences being 112.1 mm^2^ (Fig. 5a). In the dissection section where the true lumen was extremely narrowed, model A overpredicted the LOA by far compared to other sections. Figure 6b and Table 3 show that positions of the centre points of the strut ends deviated from the post-TEVAR CT scan by an average of 10.8, 11.6 and 7.1 mm in the three sections, respectively.

**Table 2:** Local open area (LOA) deviation (%) in three landing sections (mean, standard deviation and maximum values)

Landing sections	${e}_{\mathrm{LOA}}$_model A_ (%)	${e}_{\mathrm{LOA}}$_model B_ (%)	${e}_{LOA}$_model C_ (%)
Healthy aorta	14.5 ± 13.2 [− 6.2, 28.8]	− 23.4 ± 10 [− 35.3, − 8.7]	− 5.8 ± 10.8 [− 22.8, 4.7]
Entry tear	3.2 ± 6.7 [− 6.1, 9.6]	− 38.3 ± 8.5 [− 50.2, − 30]	− 3.2 ± 3.8 [− 6.7, 1]
Dissection	38.7 ± 9.3 [24, 50.8]	− 15.3 ± 9.6 [− 37, − 5.3]	− 1.7 ± 3.1 [− 9.2, 1.3]

**Table 3:** Deviation of stent strut end centre point ($e{}_{c}$) in three landing sections (mean, standard deviation and maximum values)

Landing sections	${e}_{c}$_model A_ (mm)	${e}_{c}$_model B_ (mm)	${e}_{c}$_model C_ (mm)
Healthy aorta	10.8 ± 1.9 [8.8, 13.8]	11.0 ± 1.9 [8.8, 14]	3.0 ± 1.9 [0.5, 5.5]
Entry tear	11.6 ± 0.5 [10.9, 12]	10.4 ± 1 [9.4, 11.7]	3.8 ± 1.2 [2.8, 5.3]
Dissection	7.1 ± 1.3 [5.5, 9]	5.7 ± 1.4 [4.2, 7.9]	4.6 ± 1.2 [2.3, 5.8]

By incorporating the pre-stress conditions of the aorta but neglecting the action of blood pressure on the SG inner surface, model B underpredicted LOA in all three sections (Fig. 5b) with a mean deviation of −15.3% in the dissection section, but poor results in the healthy aorta section (-23.4%) and the entry tear region (-38.3%) (Table 2 and Fig. 6a). The LOA difference on section borders was 54.1 mm^2^ between P3 and D3 and 74.3 mm^2^ for P5 and D5, which were much lower than the difference measured from the post-TEVAR CT scan. Positions of the stent struts were slightly better predicted by model B with mean values of 11.0 mm, 10.4 mm and 5.7 mm in the three landing sections (Table 3 and Fig. 6b).

Model C included both pre-stress conditions of the aorta and the action of internal pressure on SG. It produced the best results by capturing the SG configuration features at the entry tear and the dissection sections with mean LOA deviations less than 6% in all regions (Table 2 and Fig. 6a). The dramatic change of LOA at the section borders (stent struts 3 and 5) was also well captured by model C (Fig. 5c) with differences of 163.4 mm^2^ for P3 to D3 and 195.5 mm^2^ for P5 to D5. As shown in Table 3 and Fig. 6b, model C achieved the smallest deviations in stent strut position.

### Stress distribution

**Fig. 7:**
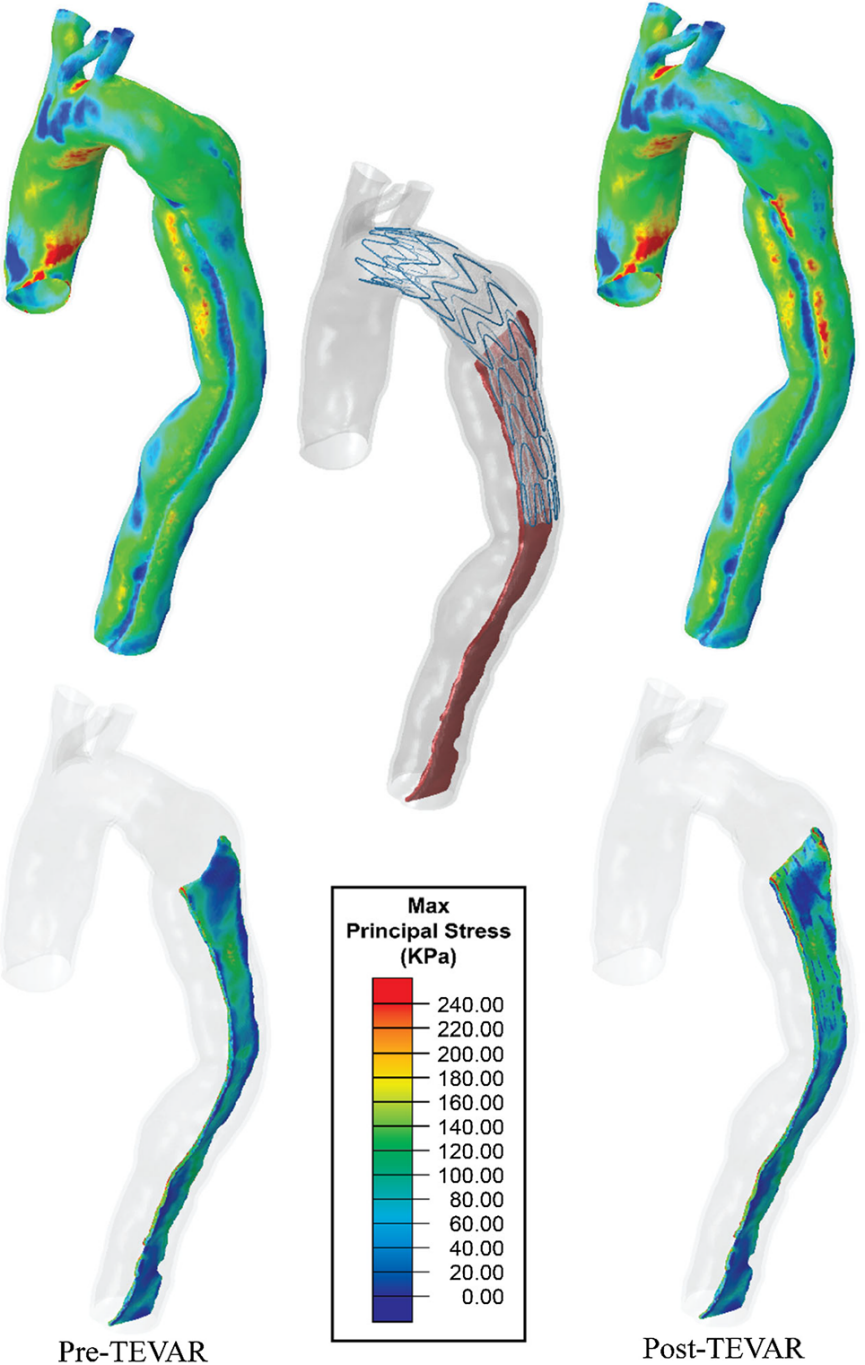
Maximum principal stress maps on the aortic wall (top) and intimal flap (bottom) in pre- and post-TEVAR models. The virtually deployed stent graft is shown in the middle with the intimal flap being highlighted. All results were obtained with model C

Figure 7 shows the maximum principal stress distributions in the aortic wall and intimal flap for the pre-TEVAR and post-TEVAR models predicted using Model C. Comparison of the pre-TEVAR and post-TEVAR stress maps suggested that SG introduced localised stress concentrations in the proximal bare metal stent landing area (Fig. 7), where the stent apexes touched the aortic wall between the left common carotid artery and the left subclavian artery. SG also introduced extra stress concentration in the dissection segment of the aorta, while the maximum principal stress in the healthy aorta and entry tear section was reduced. Table 4 shows quantitative comparison of the maximum principal stress in the three landing sections in pre- and post-TEVAR models. Following the TEVAR procedure, there was a 23% reduction in the maximum principal stress in the entry tear region and 16% in the healthy aorta. Compared to pre-TEVAR models, SG deployment increased the maximum principal stress in the dissection section by 46% on the aortic wall and 31% on the intimal flap.

**Table 4:** Maximum principal stress (KPa) in different landing sections obtained with model C (mean, 10th percentile and 90th percentile)

Landing sections		Pre-TEVAR	Post-TEVAR
Healthy aorta		103.09 [43.53, 179.43]	86.83 [34.17, 155.74]
Entry tear		105.55 [69.09, 142.46]	81.49 [49.41, 117.96]
Dissection	Aortic wall	83.65 [43.38, 129.77]	122.02 [65.61, 188.8]
	Intimal flap	54.63 [14.97, 93.26]	71.78 [14.45, 123.2]

## Discussion

Virtual SG deployment in aortic dissection poses several challenges owing to geometric complexity and mechanical nonlinearity. Unlike aortic aneurysms or coarctation of aorta, aortic dissection usually involves two lumens with multiple entry and re-entry tears. This is further complicated by the hyperelastic behaviour of dissected aorta, superelastic mechanical property of Nitinol wire along with contact nonlinearity introduced by the synthetic graft; all these bring additional challenges when modelling the interaction between SG and the local aorta. The solid–solid interaction between the aorta and SG in type B aortic dissection was investigated by Ma et al. and Meng et al. (Ma et al. 2018b; Meng et al. 2020), but these studies ignored pre-stress conditions of the aorta and pressure changes after TEVAR.

Our simulation model offers several advantages over existing methods. First, it incorporates non-uniform thickness of the intimal flap by using a purpose-built segmentation approach, allowing us to treat the intimal flap as a different material from the aortic wall. This feature is important when simulating acute and chronic stages of the pathology in the future (Peterss et al. 2016). Second, by adopting nodal specific displacement on a virtual sheath in the SG deployment simulation, our model is able to handle unusual curvatures and local narrowing in type B dissection geometry. Finally, in an attempt towards simulating the deformation of the deployed SG more accurately, our model considers the effect of pre-stressed conditions and the action of blood pressure on graft internal surface. This also enabled us to examine the influence of each of these factors on improving the simulation accuracy.

Qualitative and quantitative assessment of the simulation results was performed by comparing the predicted SG configuration with the actual configuration reconstructed from post-TEVAR CT scan. Not surprisingly, model A overpredicted LOA by far throughout the stented section due to the absence of pre-stress in the aortic wall, whereas model B significantly underpredicted LOA due to the lack of graft internal pressure. Model C produced the best results in all sections, especially in the dissection segment. With regard to positioning of the stent struts, model C also achieved the best performance.

When making quantitative assessment of the simulation accuracy, the following factors must be considered: (i) the post-TEVAR CT scan used to reconstruct the SG configuration for comparison was performed 3 months after the TEVAR procedure; (ii) the intimal flap was modelled as a linear elastic material with a Young’s modulus taken from the literature (Deplano et al. 2019); and (iii) the material parameters for the aortic wall were representative of dissected aorta but not specific to the patient included in the simulation study. Since wall remodelling begins immediately following TEVAR, it would be reasonable to expect the LOA extracted from the 3-month post-TEVAR CT scan to be slightly larger than that at the time of intervention (Conrad et al. 2009). This might explain why model C underpredicted LOA in all sections when compared with the follow-up CT scan.

Of course, the choice of material properties for the initial flap and aortic wall as well as contact conditions among the multiple components (stent, graft, intimal flap and aortic wall) will also influence the predicted SG configuration. The effects of material properties for the intimal flap and aortic wall on the predicted LOA of SG were assessed using model C. Another set of parameters for the Yeoh hyperelastic model (${c}_{10}$ = 37.1 kPa, ${c}_{20}$ = 503.9 kPa and ${c}_{30}$ = 721.4 kPa) was determined based on the anisotropic material model reported by Deplano et al. (2019), which displayed approximately twofold stiffer behaviour than the material model for the aortic wall adopted in our study. Our simulation results showed that the LOA was reduced by 5.3% when using the much stiffer hyperelastic material for the aortic wall. Similarly, there was a 5.9% reduction in LOA when the Young’s modulus for the intimal flap was increased from 277 to 350 kPa.

Auricchio et al. simulated SG deployment in a patient-specific ascending aorta with the assumption of a rigid aortic wall and achieved high accuracy in their simulation model with errors of < 1.5 mm in cross-sectional graft radius (Auricchio et al. 2013). More recently, Derycke et al. reported their simulation study on the deployment of a double branch SG in an aortic arch aneurysm, achieving a good agreement between simulation results and post-operative CT scan with diameter deviations of 3.2 ± 4.0% (Derycke et al. 2019). In our simulations of SG deployment in a patient-specific type B aortic dissection, we achieved LOA deviations of less than 6.0% (equivalent to ~ 3% in diameter) with model C. Regarding spatial deviations, model C showed deviations of up to 4.6 ± 1.2 mm, which are comparable to those reported by Derycke et al. (Derycke et al. 2019).

Including pre-stress conditions of the aorta did not alter the qualitative distribution of the maximum principal stress (MPS). Model C predicted a local high stress concentration between the left carotid artery and left subclavian artery, where the proximal end of SG landed (Fig. 7). This finding is consistent with those reported in the literature (Ma et al. 2018b; Meng et al. 2020). Comparisons of the MPS values (Table 4) suggested that following the SG deployment, there was a 23% reduction in MPS in the entry tear section where an aneurysmal enlargement was present. Considering the shielding effect of SG, this stress reduction was expected and was similar to the simulation results for aortic abdominal aneurysm (Hemmler et al. 2019a). However, SG deployment caused stress elevation in the dissection segment with MPS being 46% higher in the aortic wall and 31% higher in the intimal flap. The differential increase in the aortic wall and intimal flap can be explained by their different material properties and thickness. Furthermore, the significant increase in MPS in the dissection section is worth noting and may play a role in TEVAR-induced complications in the distal landing zone.

## Limitations

In this study, we assumed the aortic wall to have a uniform thickness with isotropic hyperelastic behaviour, rather than considering it as an anisotropic fibre-reinforced material (Roy et al. 2014). The aortic wall should also have different material properties and wall thickness in the true lumen and false lumen side due to splitting of the wall layers. The influence of different material models and parameters for the aortic wall and intimal flap should be further investigated through a comprehensive sensitivity analysis. The motion of aortic root was neglected in the current simulation. Furthermore, blood flow and its interactions with the aortic wall and SG were not modelled directly, and a constant and uniform pressure loading was applied at the graft internal surface instead, which neglected the pulsatile nature of blood flow and haemodynamic changes after TEVAR. This might have contributed to the underestimation of LOA at the P1 end which is not covered by the graft, hence not subject to the same blood pressure acting on the graft internal surface. Therefore, future studies combining computational fluid dynamics with our FEM-based SG deployment simulation with anisotropic aortic wall will be performed. Finally, only one patient-specific case was included in this study to demonstrate the proof of concept, and multiple cases will be needed for a more rigours validation.

## Conclusion

In this work, we introduced a virtual SG deployment workflow which can handle anatomical complexities encountered in type B aortic dissection. The feasibility of this new approach was demonstrated by simulating SG deployment in a complicated aortic dissection based on patient-specific information. Simulation results were compared with the post-TEVAR CT scan to assess differences in LOA and SG positioning, and a good overall agreement was achieved with model C which included both pre-stress of the aortic wall and SG internal pressure. Simulation models without applying pre-stress conditions of the aortic wall or SG internal pressure were proved to be less accurate, confirming the need to include both effects for patient-specific simulation of SG deployment in type B aortic dissection. It is hoped that the simulation model presented here can also help to understand the role of biomechanical factors in post-TEVAR complications in the future.

## References

[CR1] Auricchio F, Conti M, Marconi S, Reali A, Tolenaar JL, Trimarchi S (2013). Patient-specific aortic endografting simulation: from diagnosis to prediction. Comput Biol Med.

[CR2] Caimi A (2020). Prediction of post-stenting biomechanics in coarcted aortas: A pilot finite element study. J Biomech.

[CR3] Canaud L, Gandet T, Sfeir J, Ozdemir BA, Solovei L, Alric P (2019). Risk factors for distal stent graft-induced new entry tear after endovascular repair of thoracic aortic dissection. J Vasc Surg.

[CR4] Conrad MF, Crawford RS, Kwolek CJ, Brewster DC, Brady TJ, Cambria RP (2009). Aortic remodeling after endovascular repair of acute complicated type B aortic dissection. J Vasc Surg.

[CR5] De Bock S (2012). Virtual evaluation of stent graft deployment: a validated modeling and simulation study. J Mech Behav Biomed Mater.

[CR6] Deplano V (2019). Mechanical characterisation of human ascending aorta dissection. J Biomech.

[CR7] Derycke L, Perrin D, Cochennec F, Albertini JN, Avril S (2019). Predictive numerical simulations of double branch stent-graft deployment in an aortic arch aneurysm. Ann Biomed Eng.

[CR8] Dong ZH (2009). Retrograde type A aortic dissection after endovascular stent graft placement for treatment of type B dissection. Circulation.

[CR9] Dong Z (2010). Stent graft-induced new entry after endovascular repair for Stanford type B aortic dissection. J Vasc Surg.

[CR10] Hemmler A, Lutz B, Reeps C, Kalender G, Gee MW (2018). A methodology for in silico endovascular repair of abdominal aortic aneurysms. Biomech Model Mechanobiol.

[CR11] Hemmler A, Lutz B, Kalender G, Reeps C, Gee MW (2019). Patient-specific in silico endovascular repair of abdominal aortic aneurysms: application and validation. Biomech Model Mechanobiol.

[CR12] Hemmler A, Lutz B, Reeps C, Gee MW (2019). silico study of vessel and stent-graft parameters on the potential success of endovascular aneurysm repair Int J Numer Method. Biomed Eng.

[CR13] Holzapfel AG (2000) Nonlinear Solid Mechanics II

[CR14] Kleinstreuer C, Li Z, Basciano C, Seelecke S, Farber M (2008). Computational mechanics of Nitinol stent grafts. J Biomech.

[CR15] Ma T, Dong ZH, Wang S, Meng ZY, Chen YY, Fu WG (2018). Computational investigation of interaction between stent graft and aorta in retrograde type A dissection after thoracic endovascular aortic repair for type B aortic dissection. J Vasc Surg.

[CR16] Ma T (2018). Incidence and risk factors for retrograde type A dissection and stent graft-induced new entry after thoracic endovascular aortic repair. J Vasc Surg.

[CR17] Meng Z, Ma T, Cai Y, Liu X, Wang S, Dong Z, Fu W (2020). Numerical modeling and simulations of type B aortic dissection treated by stent-grafts with different oversizing ratios. Artif Organs.

[CR18] Nienaber CA, Clough RE (2015). Management of acute aortic dissection. Lancet.

[CR19] Pantaleo A (2016). Distal stent graft-induced new entry: an emerging complication of endovascular treatment in aortic dissection. Ann Thorac Surg.

[CR20] Pape LA (2015). Presentation, diagnosis, and outcomes of acute aortic dissection: 17-year trends from the international registry of acute aortic dissection. J Am Coll Cardiol.

[CR21] Perrin D, Badel P, Orgeas L, Geindreau C, Dumenil A, Albertini JN, Avril S (2015). Patient-specific numerical simulation of stent-graft deployment: validation on three clinical cases. J Biomech.

[CR22] Perrin D, Demanget N, Badel P, Avril S, Orgeas L, Geindreau C, Albertini JN (2015). Deployment of stent grafts in curved aneurysmal arteries: toward a predictive numerical tool Int J Numer Method. Biomed Eng.

[CR23] Perrin D, Badel P, Orgeas L, Geindreau C, du Roscoat SR, Albertini JN, Avril S (2016). Patient-specific simulation of endovascular repair surgery with tortuous aneurysms requiring flexible stent-grafts. J Mech Behav Biomed Mater.

[CR24] Peterss S (2016). Changing Pathology of the Thoracic Aorta From Acute to Chronic Dissection: Literature Review and Insights. J Am Coll Cardiol.

[CR25] Romarowski RM, Conti M, Morganti S, Grassi V, Marrocco-Trischitta MM, Trimarchi S, Auricchio F (2018). Computational simulation of TEVAR in the ascending aorta for optimal endograft selection: A patient-specific case study. Comput Biol Med.

[CR26] Romarowski RM, Faggiano E, Conti M, Reali A, Morganti S, Auricchio F (2019). A novel computational framework to predict patient-specific hemodynamics after TEVAR: Integration of structural and fluid-dynamics analysis by image elaboration. Comput Fluids.

[CR27] Roy D, Holzapfel GA, Kauffmann C, Soulez G (2014). Finite element analysis of abdominal aortic aneurysms: geometrical and structural reconstruction with application of an anisotropic material model. IMA J Appl Math.

[CR28] Vad S, Eskinazi A, Corbett T, McGloughlin T, Vande Geest JP (2010). Determination of coefficient of friction for self-expanding stent-grafts. J Biomech Eng.

[CR29] Votta E, Presicce M, Della Corte A, Dellegrottaglie S, Bancone C, Sturla F, Redaelli A (2017). A novel approach to the quantification of aortic root in vivo structural mechanics. Int J Numer Method Biomed Eng.

[CR30] Yeoh OH (1993). Some forms of the strain energy function for rubber. Rubber Chem Technol.

